# Elucidation of the life cycle of the trematode *Curtuteria arguinae* (Digenea: Himasthlidae), using environmental DNA detection methods

**DOI:** 10.1017/S0031182025100267

**Published:** 2025-06

**Authors:** Leslie Stout, Guillemine Daffe, Aurélie Chambouvet, Adrien de Montaudouin, Flore Daramy, Xavier de Montaudouin

**Affiliations:** 1 CNRS, Bordeaux INP, EPOC, UMR 5805, Station Marine, University of Bordeaux, Arcachon, France; 2 CNRS, OASU, UAR 2567 POREA, University of Bordeaux, Pessac, France; 3 CNRS, UMR 7144 AD2M, ECOMAP, Station Biologique de Roscoff, Sorbonne Université, Roscoff, France; 4 SEPANSO Aquitaine, Bordeaux, France

**Keywords:** birds, *Cerastoderma edule*, cox1, eDNA, gastropods, molecular diagnosis, morphology, parasite, SSU (18S), trematoda biology

## Abstract

Detection approaches based on environmental DNA (eDNA) are widely used for free-living species but remain underutilized for parasite species. This study applies eDNA detection methods to elucidate the life cycle of the trematode *Curtuteria arguinae*, which infects the socioeconomically and ecologically important edible cockle (*Cerastoderma edule*) as its second intermediate host along the northeastern Atlantic coast, including Arcachon Bay, France. The first intermediate and definitive hosts remained unknown. To identify these hosts – presumed to be a gastropod and a shorebird – we developed a quantitative PCR (qPCR)-based eDNA approach targeting partial *cox1* and *SSU* gene regions of *C. arguinae*. We tested for *C. arguinae* eDNA presence in water samples containing separately five dominant gastropod species and fecal samples from known cockle predators, the European oystercatcher (*Haematopus ostralegus*) and gulls (*Larus* spp.), collected in Arcachon Bay. *C. arguinae* eDNA was only detected in water containing the needle snail (*Bittium reticulatum*), with cercarial emergence confirming infection in 1.6% of individual hosts. Morphological analysis of the cercarial and metacercarial stages revealed variability in collar spine visibility. Additionally, *C. arguinae* was detected by qPCR in 42% of oystercatcher feces and no gull feces, suggesting oystercatchers are the definitive host. This study is the first to elucidate the complete life cycle of *C. arguinae*, identifying *B. reticulatum* as its first intermediate host and *H. ostralegus* as its definitive host. Our findings highlight the potential of eDNA approaches for resolving parasite life cycles and enabling advances in ecological research on *C. arguinae*.

## Graphical abstract

**Figure:**
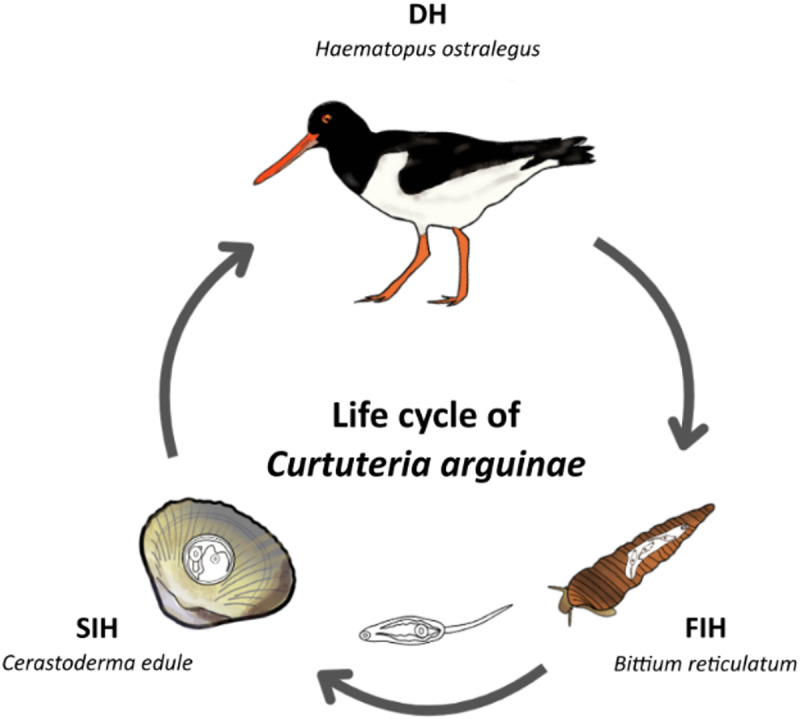

## Introduction

Integrating parasites into biodiversity and ecosystem functioning frameworks is increasingly recognized as essential, given their vital roles in ecological processes (Preston et al., 2016; Frainer et al., 2018). A critical step towards this integration involves understanding a parasite’s host range and ecological impacts, especially when a host species, such as the edible cockle, *Cerastoderma edule* Linnaeus, 1758 (Bivalvia: Cardiidae), plays a key role in ecosystem functioning. This marine bivalve is considered an ecosystem engineer that supports an economically significant shellfishery along the northeastern Atlantic coast. It provides numerous ecosystem services (Carss et al., 2020) and hosts a rich community of digenean trematodes with over 15 species in Europe and northwest Africa (de Montaudouin et al., 2021a; Stout et al., 2024). Among these parasites is the echinostome *Curtuteria arguinae* Desclaux, Russell-Pinto, de Montaudouin and Bachelet, 2006. *C. arguinae* (Digenea: Echinostomatoidea: Himasthlidae) was first described in Arcachon Bay (SW France) by Desclaux *et al*. (2006) as metacercariae infecting cockles as their second intermediate host. Subsequent observations have recorded its presence in Portugal, Morocco, and sporadically in Brittany (NW France) (de Montaudouin et al., 2009). In particular, cockle infections on Banc d’Arguin (Arcachon Bay, France) are characterized by persistently high prevalence and intensity. Stout et al. (2022) reported that 88% of adult cockles were infected by *C. arguinae* overall on Banc d’Arguin in 2021, with some individuals harbouring up to 766 metacercariae. These heavy infection loads, with metacercariae lodged in the base of the cockle’s foot, visceral mass and mantle tissue (excluding its thick margin), likely have substantial but as yet largely understudied impacts on cockle fitness and population dynamics (Desclaux et al., 2006).

Such effects are particularly concerning given the ongoing decline in cockle populations in Arcachon Bay (de Montaudouin et al., 2021b). In this context, it is interesting to explore *C. arguinae*’s effects at individual, population and ecosystem levels, for which it is first of all essential to understand its complete life cycle. The life cycle of a himasthlid trematode is complex,typically involving three hosts. Adult himasthlids sexually reproduce within a vertebrate definitive host, a bird, releasing their eggs into the environment via the host’s feces. These eggs develop into free-living miracidia larvae, which infect a first intermediate host, a mollusc, typically a gastropod. Within this host, the parasite transforms into sporocysts and rediae, asexually producing numerous cercariae larvae. Cercariae are released into the environment and rapidly infect a second intermediate host, an invertebrate, where they settle in specific tissues as metacercariae. Finally, the parasite is transmitted trophically when the definitive host preys upon the second intermediate host, completing the life cycle (Niewiadomska and Pojmańska, 2011).

Despite the initial description of *C. arguinae* nearly two decades ago in its second intermediate host, *C. edule*, the identities of the first intermediate and definitive hosts remained unknown, largely due to methodological challenges. Indeed, morphological observation of endoparasites can be complicated due to conservational constraints restraining the possibility of dissecting potential host species (mostly vertebrates), particularly in protected areas like Banc d’Arguin, a national natural reserve, or due to ecological phenomena (e.g. low infection prevalence or strong seasonality of infection), which constitute barriers for the observation of infections and the elucidation of life cycles using traditional morphological methods. Efforts to identify the first intermediate host of *C. arguinae* have, so far, been inconclusive. A survey of 6500 individuals belonging to nine gastropod species collected on Banc d’Arguin failed to identify the host (Desclaux, 2003). This may reflect a low prevalence of infection in gastropods or seasonal variation rather than host rarity, as first intermediate hosts of himasthlid species are typically abundant gastropods, such as *Peringia ulvae* for *Himasthla continua* and *H. interrupta, Littorina littorea* for *H. elongata* and *Tritia reticulata* for *H. quissetensis*. The first intermediate host of *C. arguinae* was thus suspected to also be a common gastropod species, whose abundance compensates for low infection prevalence and supports the high prevalence observed in downstream hosts, i.e. cockles. As with most himasthlid trematodes, *C. arguinae* is presumed to use shorebirds as definitive hosts. For example, the life cycle of its New Zealand congener, *C. australis* (Allison, 1979), implicates oystercatchers (*Haematopus finschi* and *H. unicolor*) as definitive hosts (Allison, 1979; Bennett et al., 2023). In Europe, the European oystercatcher (*H. ostralegus*), which is abundant on Banc d’Arguin and known to prey on cockles, was thus a plausible candidate as a definitive host of *C. arguinae* (e.g. Sutherland, 1982; Triplet, 1996). Banc d’Arguin also hosts a substantial colony of gulls (*Larus* spp.), known as predators of cockles as well (Norris et al., 2000), representing other plausible definitive hosts for the parasite. To address the challenges hampering host identification, molecular tools such as environmental DNA (eDNA) provide a powerful approach for detecting elusive parasite life stages under complex conditions. Widely used for studying free-living aquatic organisms, eDNA approaches are increasingly being applied to trematodes (Bass et al., 2015, 2023; Huver et al., 2015; Rusch et al., 2018; Thomas et al., 2022). In parasitology, the scope of eDNA is large, including DNA extracted from environmental samples such as water, soil, sediment or air, but also biological material from host species, such as feces, blood or tissue. (Bass et al., 2023). It thus represents a sensitive and adaptable tool that enables the detection of parasite DNA in host tissue or nonintrusively in water, sediment or fecal samples (Honma et al., 2011; Bass et al., 2015, 2023; Cabodevilla et al., 2024).

In this study, we developed *C. arguinae*-specific primers to detect *C. arguinae* eDNA by quantitative PCR (qPCR) in water and bird fecal samples, aiming to identify both its first intermediate and definitive hosts. We described the morphological features of *C. arguinae* cercariae, with a special focus on the circumoral collar spines. Indeed, the presence and the number of spines are key for identifying echinostome species, with *C. arguinae* displaying 33 spines. However, the existence of a delayed appearance of collar spines has been reported in another echinostome, *Isthmiophora hortensis* (Sohn et al., 2024), prompting us to investigate whether a similar process occurs in *C. arguinae*. Finally, this study elucidates the complete life cycle of *C. arguinae* and demonstrates the efficacy of an eDNA-based approach in achieving this goal.

## Materials and methods

### Study area

Gastropods, cockles and bird feces were sampled on Banc d’Arguin (44.60°N, 1.25°W), Arcachon Bay, France. Arcachon Bay is a 182-km^2^ semienclosed macrotidal lagoon on the southwestern Atlantic coast of France, connected to the Atlantic Ocean by a 24-km^2^ wide channel where Banc d’Arguin is located. This 4 km × 2 km sand bank (at low tide) is a national nature reserve comprising sand dunes and semisheltered sandflats where various marine bird species winter, nest or rest during migrations. Secondarily, cockles were collected on Ile aux Oiseaux (44.70°N, 1.17°W, Arcachon Bay, France), an island encompassing 17 km^2^ of predominantly muddy and sandy intertidal flats at low tide.

### Design of *Curtuteria arguinae*-specific primers

Two primer pairs were designed to specifically amplify *Curtuteria arguinae* DNA: one targeting a fragment of the mitochondrial cytochrome *c* oxidase I gene (cox1) and the other a fragment of the small subunit (*18S*) ribosomal RNA gene (*SSU*). Suitable sequences were identified through two multiple sequence alignments of 94 *cox1* sequences and 126 *SSU* sequences, respectively, from previous studies (de Montaudouin et al., 2021a; Stout et al., 2024). The dataset included 17 genetic lineages of trematodes infecting cockles, incorporating sequences from *C. arguinae* and closely related species of the superfamily Echinostomatoidea (Stout et al., 2024). Candidate primers specific to *C. arguinae* were identified in variable regions within the alignment. Primer specificity was first verified *in silico* using Primer-BLAST (NCBI). Second, experimental validation followed by testing with mock communities including (1) a mix containing genomic DNA (gDNA) from 12 trematode species infecting cockles (included in the multiple sequence alignments), including *C. arguinae* and (2) the same mix excluding *C. arguinae* DNA. All gDNA samples were obtained as described by Stout et al. (2024). Thermocycling conditions were optimized to ensure specific amplification of *C. arguinae* gDNA, whether from pure monospecific gDNA samples, the mock community 1 (as described earlier) or whole individuals of *C. edule* previously identified as infected by *C. arguinae* (under a stereomicroscope Nikon SMZ1500). Initial primer validation was performed via PCR, followed by quantitative PCR (qPCR) using a LightCycler 480 II (Roche). Two primer pairs yielding exclusive amplification of *C. arguinae* DNA were retained. For *cox1*, CACOIdetF (5ʹ-CGGGAGTCGTGCTCGTTTAT-3ʹ) and CACOIdetR (5ʹ-TGCGCTACCACAAACCAAGT-3ʹ) amplified a fragment of 147 bp. For *SSU*, CA18SdetF (5ʹ-TTACGGCCGGGTCAAACTC-3ʹ) and CA18SdetR (5ʹ-CCATACAAATGCCCCCGTCT-3ʹ) amplified a fragment of 302 bp. To verify the sensitivity of the primers for detecting *C. arguinae* eDNA, the limit of detection (LOD) was determined for both the *cox1* and *SSU* primer sets. LOD testing followed recommendations of Hou et al. (2010) and was based on 10-fold serial dilutions of a linear DNA standard (purified PCR amplicons of plasmid DNA), which were amplified in triplicate using qPCR. The estimated LOD for the *cox1* primers was 2.5 × 10^3^ copies µL^−1^, with a mean cycle threshold (Ct) value of 33. For the *SSU* primers, the LOD was lower, at 2.5 × 10^1^ copies µL^−1^, with a mean Ct value of 35. Additionally, gDNA extracted from a pool of 10 *C. arguinae* cercariae was diluted 1:100 and amplified by qPCR in triplicate. The assays were able to detect DNA equivalent to 0.1 cercaria, with mean Ct values of 30 (cox1) and 26 (*SSU*), confirming that both primer sets presented high sensitivity.

### Identification of the first intermediate host

#### Sample collection and experimental setting

**Figure 1.**
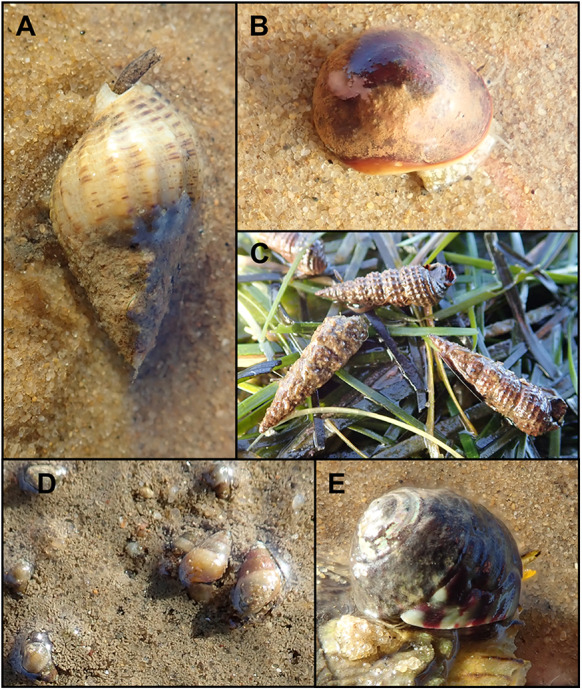
Figure panel of the different species of marine gastropods sampled on Banc d’Arguin (Arcachon Bay, France). (A) *Tritia reticulata*; (B) *Tritia neritea*; (C) *Bittium reticulatum*; (D) *Peringia ulvae* and (E) *Steromphala umbilicalis*. Scale bars represent 1 cm.

Marine gastropods were collected at low tide in and around *Zostera noltei* seagrass patches on Banc d’Arguin, in an area where cockle infection by *C. arguinae* is high (median: 102 metacercariae per cockle, Stout et al., 2022). Five dominant gastropod species were collected: *Tritia reticulata* (Linnaeus, 1758, Nassariidae), *T. neritea* (Linnaeus, 1758, Nassariidae), *Peringia ulvae* (Pennant, 1777, Hydrobiidae), *Bittium reticulatum* (da Costa, 1778, Cerithiidae) and *Steromphala umbilicalis* (da Costa, 1778, Trochidae) (Figure 1). The 250 largest individuals of each species were placed in decontaminated plastic containers with 50 individuals per container (five containers per species). Exceptionally, due to its patchy distribution influenced by hydrodynamics (Armonies and Hartke, 1995), only 130 individuals of *P. ulvae* could be collected and were distributed among four containers. One additional decontaminated container, free of gastropods, was set up as a negative control. All containers were filled with 1 L of open-ocean surface seawater, unlikely to contain *C. arguinae* DNA, and maintained at room temperature (23.5 °C) under natural light conditions.

#### Water filtration and eDNA extraction

After 24 h, water was filtered through a 3.0-µm pore size polycarbonate filter of 142-mm diameter (Isopore membrane filter TSTP14250, Merck) using a peristaltic pump to capture potential extracellular DNA or cercariae of *C. arguinae* that were shed into the water. Filter funnels, tubes and forceps used to handle the filters were decontaminated by UV before use, washed with 10% bleach and rinsed with ultrapure water (Milli-Q, Merck) before and between samples. After filtration, each filter was cut in half with a sterile razor blade. One half was stored at −20 °C as a backup and the other was processed for DNA extraction and stored overnight in 360 µL of Buffer ATL (from the Qiagen DNA extraction kit) at room temperature.

#### Molecular analyses

gDNA was extracted using the DNeasy Blood & Tissue Kit (Qiagen), with doubled reagent volumes from step 1 to step 3 due to the high sample load and a 2-h incubation for the lysis. Therefore, step 4 was repeated 2–3 times to allow all of the mixture to be centrifuged through the spin column. Subsequent steps were performed following the manufacturer’s instructions. Finally, a few filtration replicates containing *T. reticulata* and *S. umbilicalis* were particularly loaded and thus subdivided into 2 separate tubes for DNA extraction, after which their resulting DNA samples were pooled. Molecular detection was performed via qPCR using CACOIdetF/CACOIdetR and CA18SdetF/CA18SdetR (Table 1). qPCR reactions were performed in a LightCycler 480 II (Roche) with optical settings for SYBR Green I. Each 20-µL reaction contained 10 µL of 2× GoTaq qPCR Master Mix (Promega), 3 µL of nuclease-free water and 5 μL of template DNA. A negative control (nuclease-free water) and a positive control (*C. arguinae* DNA) were included for every qPCR assay. Cycling conditions for each primer set are detailed in Table 2. Melt curves were produced after the amplification cycles to check the melting temperature (T_m_) of the qPCR products. Only samples with a cycle threshold (Ct) value below 35 were considered positive.

**Table 1.** Primers used for the detection or sequencing of *Curtuteria arguinae* and their specific cycling conditions

Name	Sequence (5ʹ → 3ʹ)	Gene	Specificity	PCR/qPCR cycling conditions	Reference
CACOIdetF	CGGGAGTCGTGCTCGTTTAT	*cox1*	*Curtuteria arguinae*	94 °C/2 min – (94 °C/15 s – 66 °C/1 min 30 s) × 40 cycles – 94 °C	Present study
CACOIdetR	TGCGCTACCACAAACCAAGT				
CA18SdetF	TTACGGCCGGGTCAAACTC	*SSU*	*Curtuteria arguinae*	94 °C/2 min – (94 °C/15 s – 66 °C/1 min 30 s) × 40 cycles – 94 °C	Present study
CA18SdetR	CCATACAAATGCCCCCGTCT				
TremCOIS2	TGTTYTTTAGKTCTGTKAC	*cox1*	Trematodes	95 °C/10 min – (95 °C/60 s – 43 °C/30 s – 72 °C/60 s) × 40 cycles – 72 °C/10 min – 16 °C	Magalhães et al. (2020)
TremCOIAS2	AATGCATMGGRAAAAAACA

**Table 2.** Detection of *Curtuteria arguinae* by quantitative PCR from the filtered water samples in which candidate first intermediate host species (gastropods) were held. Results (cycle threshold value [Ct]/melting temperature [°C]) are shown for the 2 gene markers (*cox1* and *SSU*)

Candidate host species	*Peringia ulvae*		*Tritia neritea*		*Tritia reticulata*		*Bittium reticulatum*		*Steromphala umbilicalis*	
Gene	cox1	SSU	cox1	SSU	cox1	SSU	cox1	SSU	cox1	SSU
Replicate 1	–	–	–	–	–	–	28/82.7	23/89.4	–	–
Replicate 2	–	–	–	–	–	–	25/82.6	21/89.5	–	–
Replicate 3	–	–	–	–	–	–	28/83.4	25/89.5	–	–
Replicate 4	–	–	–	–	–	–	27/83.6	25/89.7	–	–
Replicate 5	/	/	–	–	–	34/89.5	32/82.9	28/89.2	–	–

#### Collection of cercariae and rediae for morphological and molecular description

Following the qPCR results, individuals of the putative first intermediate host were collected in October and November 2023 in the same sampling area on Banc d’Arguin using a 2-mm mesh sieve. In the laboratory, 991 individuals were placed individually in plastic boxes (dimensions: 5.5 cm × 5.5 cm × 2.5 cm) filled with seawater (salinity 30) and kept at a temperature of approximately 22 °C under light conditions for 24 h to induce cercarial emergence. Each box was examined under a stereomicroscope (Wild Heerbrugg 1985 Inv. 2380). Cercariae were collected in sterile 1.5-mL tubes and stored in 96% ethanol at −20 °C until further analysis. Ethanol-fixed specimens were examined under a Nikon SMZ25 stereomicroscope and a Nikon Eclipse Ci microscope, and photographed with a Nikon DS-Ri 2 camera. Measurements were performed on 15 ethanol-fixed cercariae under coverslip pressure using the NIS-Elements Analysis software. The drawing of the cercaria was made using the vector graphics softwares InkScape (1.2.2, retrieved from https://inkscape.org) and Affinity Designer 2 (v2.4.2, retrieved from https://affinity.serif.com/fr/designer/). Dehydrated specimens used for examination by scanning electronic microscopy (SEM) were prepared by critical point drying, coated with gold and examined and photographed with a Hitachi TM3030.

DNA sequences were generated for three samples containing a pool of 10 cercariae shed by the first intermediate individual host, conserved in 96% ethanol. gDNA was extracted using the DNeasy Blood and Tissue kit (Qiagen), following the manufacturer’s instructions. A fragment of the *cox1* gene was amplified by PCR using the primers TremCOI2S/TremCOI2AS (Magalhães et al., 2020). A negative control (nuclease-free water) was included for every PCR reaction. All PCR amplification reactions were performed in a 50-μL total volume using GoTaq G2 Flexi DNA polymerase (Promega), following the manufacturer’s protocol with 1 μL diluted template DNA. Cycling conditions are detailed in Table 1. Amplified PCR products were checked on a 1% agarose gel stained with ethidium bromide. PCR amplifications were sent for Sanger sequencing to Macrogen Europe B.V. Consensus sequences were assembled and manually edited using the MEGA v11.0.13 software (Tamura et al., 2021). Three sequences were deposited in GenBank under accession numbers PV216840–PV216842. Sequences were compared in MEGA v11.0.13 using the *p-*distance for genetic divergence calculations. Reference sequences included previously published partial *cox1* and *SSU* sequences of *C. arguinae* obtained from metacercariae infecting cockles (GenBank accession numbers PP987234–PP987239, MT002920).

#### Experimental infection of cockles by cercariae

Ten infected snails were placed individually in plastic containers containing seawater at 24 °C under light conditions to stimulate cercarial emergence. Small, i.e. young, cockles (10–14 mm, i.e. about a year old) harbouring little to no *C. arguinae* metacercariae were collected at Ile aux Oiseaux (Arcachon Bay, France), where the prevalence and parasite abundance are almost null (de Montaudouin et al., 2012 and pers. obs.). Once *C. arguinae* cercariae had emerged (after approximately 7 h), 4 cockles were placed in each box and kept under light conditions at 24 °C. One cockle per box was checked for *C. arguinae* metacercariae after 1, 2, 3 and 6 days. Metacercariae were examined for the presence of circumoral collar spines under a stereomicroscope (Nikon SMZ25) and/or a light microscope (Nikon Eclipse Ci).

### Definitive host

#### Sample collection

Fresh feces from European oystercatchers (*Haematopus ostralegus*) and gulls (*Larus* spp.) were collected in winter and spring from December 2022 to February 2024 on Banc d’Arguin. Oystercatcher feces were sampled on the beach, while gull feces were collected near nests. Samples were stored in 5-mL tubes at 4 °C.

#### Molecular analyses

gDNA was extracted from approximately half of each sample using the DNeasy PowerSoil Pro kit (Qiagen) following the manufacturer’s instructions. *C. arguinae* DNA detection was performed using the primers CA18SdetF/CA18SdetR via qPCR as previously described (Table 1). Positive qPCR results prompted further examination of the remaining part of the fecal samples for the presence of trematode eggs under a stereomicroscope (Nikon SMZ1500), using a pipette and diluting the sample in seawater, following Born-Torrijos et al. (2017). Potential eggs were collected and stored individually in 1.5 mL tubes at −20 °C. DNA was extracted using the DNeasy Blood and Tissue kit (Qiagen), with a slightly increased amount of Proteinase K (25 µL, 600 mAU mL^−1^) to facilitate eggshell lysis.

## Results

### First intermediate host

#### Identification of the first intermediate host by eDNA detection

Out of 25 water samples tested for the presence of *Curtuteria arguinae*, five tested positive with both primer pairs targeting partial sequences of the *cox1* and *SSU* encoding genes by qPCR (Table 2). All positive samples were from gDNA recovered from filtered water containing needle snails, *Bittium reticulatum*. These samples exhibited significant fluorescence with cycle threshold (Ct) values ranging from 25 to 32 using the *cox1* primers and 21 to 28 using the *SSU* primers, with mean T_m_ values of 83.0 °C and 89.5 °C, respectively (Table 2). Additionally, one water sample containing *Tritia reticulata* (replicate 5) showed fluorescence with a Ct value of 34 but remained entirely negative with the *cox1* primers. Therefore, it was not considered a reliable positive result but rather a false positive due to late nonspecific amplification or potential contamination. The control sample, along with the remaining samples associated with the gastropods *T. reticulata, T. neritea, Peringia ulvae* and *Steromphala umbilicalis*, showed no presence of *C. arguinae* eDNA. Taken together, these results identified, for the first time and using eDNA detection, the needle snail, *B. reticulatum*, as the putative first intermediate host of the trematode *C. arguinae*.

#### Morphological features of rediae and cercariae

Out of 991 individuals collected on Banc d’Arguin using a 2-mm mesh sieve, a total of 16 needle snails were identified as infected through cercarial emergence. This corresponded to a prevalence of 1.6% for this shell size class and considering cercariae-shedding snails. The shell height of infected snails ranged from 9.8 to 11.9 mm, with a mean of 10.7 mm.

**Figure 2.**
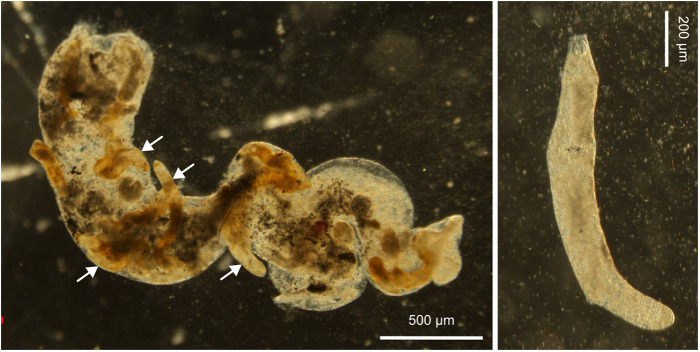
Microphotographs of rediae of *Curtuteria arguinae* infecting *Bittium reticulatum* tissue. Left: *B. reticulatum* tissue sample (digestive glands and gonads) with apparent rediae (white arrows). Right: closer view of a redia.

**Figure 3.**
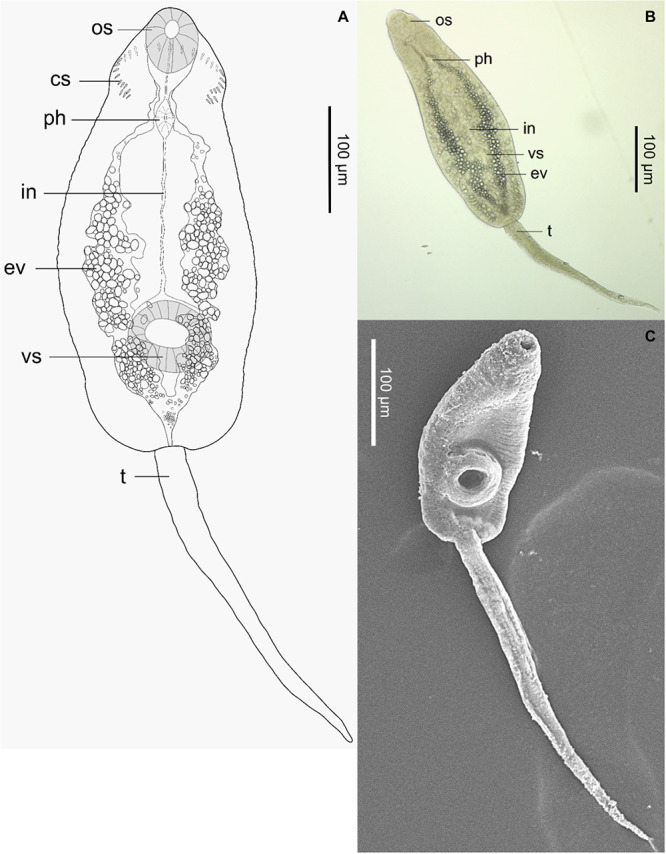
Figure panel of cercariae of *Curtuteria arguinae*. (A) Drawing with the 33-spine circumoral collar: os, oral socker; cs, collar spine; ph, pharynx; in, intestines; ev, excretory vesicles; vs, ventral sucker; t, tail. (B) Photograph under a stereomicroscope. (C) SEM microphotograph.

Optical observation of these samples under a stereomicroscope revealed that rediae were located towards the posterior end of the gastropod’s body, around the digestive glands and gonads (Figure 2). Rediae had a terminal mouth, a well-distinguishable, rounded pharynx, and were elongated, varying in length. They contained cercariae at various stages of maturity, some of which were also found free outside the rediae. Emerged cercariae were highly active, moving rapidly with their tails and performing contracting movements with their bodies upon reaching the bottom. The cercarial body was elongated, slender and slightly flattened dorsoventrally, with its region just above the ventral sucker. Body length ranged from 250 to 400 µm, with a width of 110–130 µm at its largest point (Figure 3, Table 3). The tail was approximately the same length as the body, measuring between 270 and 370 µm. The body tegument bore folds along its entire surface. The oral sucker measured 50–80 µm in length and 40–70 µm in width, featuring an oval aperture surrounding the mouth, which led to an oval pharynx. The oesophagus was elongated, bifurcating close to or above the large ventral sucker. The latter was 50–80 µm in length and 50–70 µm in width and protuberant. The excretory system was clearly visible, extending from the oral sucker to the base of the tail without lateral diverticula. It was filled with dense, dark excretory granules, obscuring the intestines. Interestingly, circumoral collar spines were not constantly visible by stereo- and light microscopy and were absent in SEM, where only external features could be observed. Some cercariae exhibited circumoral collar spines, while others with the identical morphology lacked them. When present, a total of 33 spines surrounded the cephalic region. These included an uninterrupted main dorsal row of 27 spines, along with 3 additional, slightly shorter angular spines on each collar corner located on the ventral side, arranged identically to the metacercarial stage.

**Table 3.** Dimensions of the main morphological features of *Curtuteria arguinae* cercariae (measurements based on 15 ethanol-fixed cercariae under coverslip pressure). Values represent the min–max (mean) measurements in µm

Morphological structure	Size (µm)
Body length	250–400 (331)
Body width	110–130 (124)
Oral sucker diameter	50 × 40–80 × 70 (53 × 48)
Ventral sucker diameter	50 × 50–80 × 70 (60 × 89)
Tail length	270–370 (345)
Number of collar spines (when visible)	33

#### Collar spines

**Figure 4.**
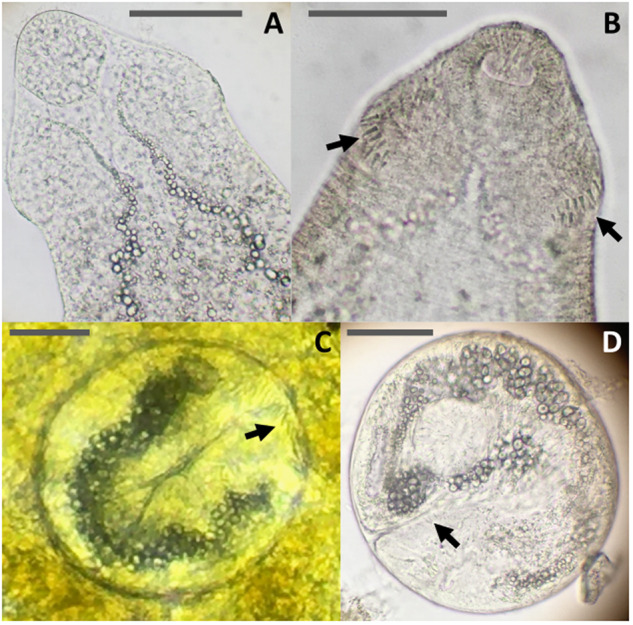
Figure panel of different larval stages of *Curtuteria arguinae* (cercariae and metacercariae), with a focus on the circumoral collar spines, present or absent, under stereo- and light microscopy. Arrows indicate the presence of spines. Scale bars represent 50 µm. (A) Cercaria without spines. (B) Cercaria with spines. (C) Metacercaria with spines encysted in the digestive glands of a cockle observed under a stereomicroscope. (D) Metacercaria with collar spines under light microscopy after extraction from the host tissue.

Cercariae of *C. arguinae* emerged from needle snails did not all exhibit the 33-spine circumoral collar observed in metacercariae. For the same cercariae-shedding needle snail, both cercariae with 33-spine collars (Figure 4B) and cercariae without spines (Figure 4A) were observed. Experimental infection of cockles with *C. arguinae* cercariae allowed for the observation of spine development at the metacercarial stage. While 0–3% of metacercariae bore collar spines up to 3 days post-infection (Figure 4D), 68% bore spines after 6 days post-infection (Figure 4C, Table 4). These results showed a clear increase of spine-bearing metacercariae over time post-infection.

**Table 4.** Total number of metacercariae of *Curtuteria arguinae* encysted in cockles by experimental infection and percentage of metacercariae for which a collar spine was visible 1, 2, 3 and 6 days after infection

	1 day	2 days	3 days	6 days
Total number of metacercariae	61	57	104	74
Metacercariae with collar spines	3%	0%	3%	68%

#### Molecular results

Partial sequencing of the *cox1* gene marker from emerged cercariae generated three sequences of 253 nucleotides (Table 5). Two sequences were highly similar if not identical to the five reference sequences obtained from metacercariae infecting cockles, with pairwise genetic divergences (*p*-distances) close to zero (0–0.4%, 0–2 nt) (Table 6). This confirmed that *C. arguinae* infects the needle snail as its first intermediate host in the form of rediae, which produce cercariae. However, a third sequence obtained from cercariae shed by a needle snail presented significant genetic divergence (12.0–13.5%) (Table 6). A BLASTn search revealed no closer sequences than the *C. arguinae* reference sequences.

**Table 5.** *Cox1* partial sequences deposited in GenBank with corresponding accession number

Sequence ID	Primers	Host	GenBank accession numbers
B2_trem_COI_TremCOI_S2	TremCOI2S/TremCOI2AS	*Bittium reticulatum*	PV216840
B3_trem_COI_TremCOI_S2		*B. reticulatum*	PV216841
B4_trem_COI_TremCOI_S2		*B. reticulatum*	PV216842

**Table 6.** Pairwise genetic distances (*p*-distances) between the partial *cox1* gene sequences obtained from cercariae shed by *Bittium reticulatum* and reference *Curtuteria arguinae* sequences

Sequence	(1)	(2)	(3)	(4)	(5)	(6)	(7)	(8)	(9)
(1) B3_trem_COI_TremCOI_S2	–	–	–	–	–	–	–	–	–
(2) B2_trem_COI_TremCOI_S2	0.135	–	–	–	–	–	–	–	–
(3) B4_trem_COI_TremCOI_S2	0.129	0	–	–	–	–	–	–	–
(4) *C. arguinae* (PP988234)	0.120	0.004	0.004	–	–	–	–	–	–
(5) *C. arguinae* (MT002920)	0.125	0	0	0.004	–	–	–	–	–
(6) *C. arguinae* (PP988236)	0.125	0	0	0.004	0	–	–	–	–
(7) *C. arguinae* (PP988237)	0.125	0	0	0.004	0	0	–	–	–
(8) *C. arguinae* (PP988238)	0.125	0	0	0.004	0	0	0	–	–
(9) *C. arguinae* (PP988239)	0.125	0	0	0.004	0	0	0	0	–

### Definitive host

A total of 160 bird feces were collected and analysed, including 108 samples from oystercatchers and 52 samples from gulls (*Larus* spp.). *C. arguinae* molecular detection resulted in 46 (43%) positive oystercatcher fecal samples and no positive gull fecal samples (0%). PCR amplification of DNA from isolated eggs was not successful due to DNA extraction failure and scarceness of potential eggs. Consequently, molecular sequences from the eggs could not be compared to existing sequences of *C. arguinae*.

## Discussion

**Figure 5.**
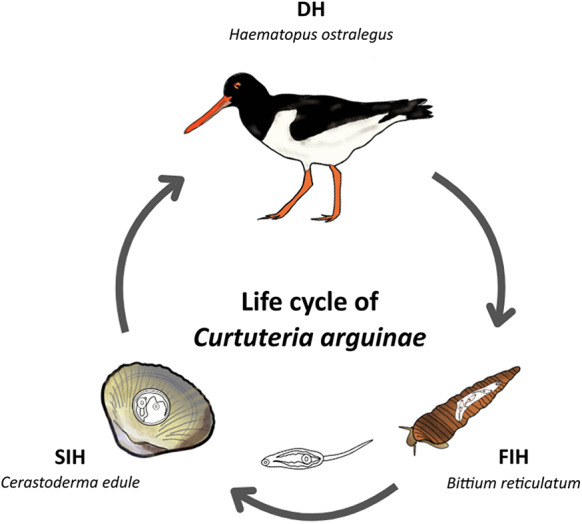
Scheme of the putative life cycle of *Curtuteria arguinae*. FIH, first intermediate host: *Bittium reticulatum* (needle snail); SIH, second intermediate host: *Cerastoderma edule* (edible cockle); DH, definitive host: *Haematopus ostralegus* (European oystercatcher). The adult worm, the miracidium and the eggs are not represented as their morphologies remain unknown.

According to the molecular and morphological results, *Curtuteria arguinae* infects needle snails *B. reticulatum* as its first intermediate host. The genetic signature of *C. arguinae* exclusively retrieved in oystercatcher feces strongly suggests that adult worms use this shorebird as a definitive host. Our results provide, for the first time, a clearer understanding of the parasite’s life cycle. The proposed life cycle for *C. arguinae* is represented in Figure 5. Interestingly, the *cox1* partial sequences retrieved from cercariae shed by needle snails also revealed cercariae with greater genetic divergence (12.0–13.5%) (sequence B3_trem_COI_TremCOI_S2) than that observed among currently available *C. arguinae* reference sequences. This level of divergence exceeds the intraspecific variation reported for the same *cox1* fragment in this species (Stout et al., 2024) and also exceeds that of its congener *C. australis* (Donald and Spencer, 2016). *C. australis* has previously been shown to encompass a cryptic species, *Curtuteria* sp. A (Leung et al., 2009), which presents 22% of genetic divergence from *C. australis* based on *cox1* sequences (Leung et al., 2009; Donald and Spencer, 2016). In the case of *C. arguinae*, the genetic divergence of 12.0–13.5% is lower than for *C. australis*, keeping in mind that the *cox1* fragment sequenced here is much shorter (259 bp vs 920 bp). Also, no morphological differences were observed among *Curtuteria* cercariae that emerged from needle snails in our study. Thus, while current data do not allow for conclusive statements about the presence of a cryptic species within *C. arguinae*, the observed genetic divergence – paired with a lack of corresponding morphological differentiation – warrants further investigation using longer gene regions and much broader sampling.

### First intermediate host: *Bittium reticulatum*

Free-living miracidia hatched from eggs infect the parasite’s first intermediate host, the needle snail (*Bittium reticulatum*). Cercarial emergence was used as a proxy for needle snail infection. Prevalence in needle snails was low (1.6%) yet expected. Indeed, the prevalence was comparable to what is typically observed (by cercarial emission or dissection) in first intermediate hosts for various trematode species, such as 2.6–3.2% for *C. australis* in *Cominella glandiformis* (Allison, 1979; Donald and Spencer, 2016), 2.4% for *Himasthla elongata* in *Littorina littorea* or 1.9% for *H. continua* in *Peringia ulvae* (Thieltges et al., 2006). However, cercarial emergence (as performed in the present study) notoriously underestimates infection rates (Born-Torrijos et al., 2014). A strong seasonality of cercarial emergence is also suspected. We therefore assume that the real prevalence in needle snails is higher. Given the high host specificity typically exhibited by digenean trematodes at this stage (Poulin, 2007), we hypothesize that needle snails are the preferred, if not the exclusive, first intermediate host of *C. arguinae* within the Banc d’Arguin ecosystem, similar to *C. australis* infecting *C. glandiformis* (Table 7). The morphological features of cercariae were consistent with those of the Himasthlidae family, such as a body not subdivided into regions of different shapes, a smaller oral sucker than the large ventral sucker that is muscular and, when apparent, a reniform head collar bearing spines with angle spine-groups (Kostadinova, 2005). Cercariae also shared general characteristics with the metacercarial stage of *C. arguinae* (Desclaux et al., 2006). There are few morphometric data available overall in the *Curtuteria* genus. Cercariae appeared to be slightly smaller than those of *C. australis* (Table 7). Comparison with the other three *Curtuteria* species, namely *C. numenii, C. grummti* and *C. haematopodis*, is not possible as they have only been described at the adult stage extracted from their definitive host, leaving their life cycle and cercariae unknown (Table 7). Interestingly, we observed both spine-bearing and spine-lacking cercariae of *C. arguinae*. The number and arrangement of collar spines in echinostome trematodes serve as an important morphological character for species identification (Table 7). The presence of 33 spines in *C. arguinae* metacercariae is a key feature that differentiates it from other himasthlid metacercariae infecting cockles, such as *H. continua, H. quissetensis* and *H. elongata*, which display 29–31 collar spines (de Montaudouin et al., 2009). However, experimental infection of cockles with cercariae revealed that the majority of metacercariae lacked visible collar spines during the initial days post-infection, with spines appearing after 6 days in over two-thirds of metacercariae. These results indicate that the collar spines develop as the larval stages mature. Interestingly, 3% of metacercariae exhibited spines as early as 1–3 days post-infection. We hypothesize that these originated from a minority of cercariae more mature at the time of emergence from the needle snail, underlining the variability in the maturity of cercariae within the first intermediate host. Some cercariae have developed visible spines already and transformed to metacercariae with spines immediately, while others need to mature further after infecting cockles before presenting spines. These observations align with reports documenting the absence of collar spines in the cercarial and early metacercarial stages of other echinostome species. The review from Fried et al. (2009) highlighted contrasting observations regarding the presence of collar spines in conspecific cercariae, although the authors suggest that these discrepancies might stem from methodological inaccuracies in light and SEM microscopy. More recently, Sohn et al. (2024) demonstrated that the collar spines of the freshwater echinostome *Isthmiophora hortensis*only develop in 24-h-old metacercariae, while earlier studies were inconsistent, observing cercariae of the same species both with and without spines. Here, we produce more evidence supporting this phenomenon.

**Table 7.** Overview of the known life cycles and morphometrics of cercariae of trematode species of the genus *Curtuteria*

	*C. arguinae*	*C. australis*	*C. numenii*	*C. grummti*	*C. haematopodis*
First intermediate host	*Bittium reticulatum* (Cerithiidae) [1]	*Cominella glandiformis* (Cominellidae) [6]	–	–	–
Second intermediate host	*Cerastoderma edule* (Cardiidae) [2,3] *Ruditapes phillipinarum* (Veneridae) [4,5] *R. decussatus* [4] *Polititapes aureus* (Veneridae) [4]	*Austrovenus stutchburyi* (Veneridae) [6] *Macomona liliana* (Tellinidae) [6,7] *Semele australis* (Semelidae) [6]	–	–	–
Definitive host	*Haematopus ostralegus* [1]	*H. finschi* [6] *H. unicolor* [8] *Larus* spp. [8,9]	*Numenius phaeopus* [10]	*Somateria mollissima* [11]	*H. ostralegus* [12]
Number of collar spines	33 [1,2]	31 [6]	29 [10]	29 [11]	33 [12]
Cercarial body length (mm)	0.3 [1]	0.69 [6]	–	–	–
Cercarial body width (mm)	0.12 [1]	0.26 [6]	–	–	–
Cercarial tail length (mm)	0.35 [1]	0.49 [6]	–	–	–
Cercarial oral sucker diameter (mm)	0.05 [1]	0.05 [6]	–	–	–
Cercarial ventral sucker diameter (mm)	0.075 [1]	0.15 [6]	–	–	–
Material fixation	Ethanol-fixed, under coverslip pressure [1]	Alive, under coverslip pressure [6]	–	–	–

References: [1] present study; [2] Desclaux et al. (2006); [3] de Montaudouin et al. (2021a); [4] Dang et al. (2009); [5] Alfeddy et al. (2024); [6] Allison (1979); [7] Leung and Poulin (2008); [8] Bennett et al. (2023); [9] McFarland et al. (2003); [10] Reimer (1963); [11] Odening (1963); [12] Smogorzhevskaya and Iskova (1966).

### Second intermediate host: *Cerastoderma edule*

Released cercariae will most likely survive 24–48 h during which they swim actively the first hours to infect the second intermediate host (de Montaudouin et al., 2016; Bommarito et al., 2020), where they encyst to form metacercariae. Infections of *C. arguinae* metacercariae have been reported in cockles on several occasions and in multiple sites in France, Portugal and Morocco (de Montaudouin et al., 2009). Interestingly, there are also a few reports of infections in other bivalves (Table 7). Dang et al. (2009) found three clam species (*Ruditapes decussatus, R. phillipinarum* and *Polititapes aureus*) infected by 0.1–1.2 metacercariae of *C. arguinae* per host overall in Arcachon Bay. On Banc d’Arguin specifically, only *R. decussatus* was reported infected by a mean of 0.7 metacercariae, while cockles were infected by a mean of 8.1 metacercariae. Furthermore, in the Oualidia lagoon (Morocco), *C. arguinae* metacercariae were found in *R. decussatus* with a prevalence of 60% and a mean abundance of 5.5 metacercariae per clam in the intertidal area in 2012 (X. de Montaudouin unpublished data). At the same site, cockles are infected by up to 100 metacercariae per host (Alfeddy et al., 2024), with 100% prevalence (Correia et al., 2020). To sum up, *C. arguinae* has been observed in four different second intermediate host species, all of which are bivalves. This relatively broad host range indicates that the parasite is not highly host-specific at this stage, a common trait among digeneans (Poulin, 2007) and similar to what is observed for *C. australis* (Table 7). However, the general prevalence and abundance being remarkably lower in the clam species compared to cockles, the status of these clams appears as rather accidental yet compatible hosts, as cercariae transformed into metacercariae, but may also be dead-ends if the definitive host cannot predate them due to their deep-burrowing behaviour. We hypothesize that they serve as facultative second intermediate hosts capable of transmitting the parasite to its definitive host, while cockles serve as the main second intermediate host due to their higher prevalence and intensity of *C. arguinae* infections. Indeed, from an ecological and evolutionary point of view, cockles appear more interesting for the parasite’s life cycle, constituting easier prey for birds (located just below the sediment surface and often at high densities) and more heavily parasitized, promising higher successful transmission rates than the clams.

### Definitive host: *Haematopus ostralegus*

Finally, the European oystercatcher (*Haematopus ostralegus*) becomes infected by consuming cockles (and optionally clams) that harbour metacercariae of *C. arguinae*. Indeed, 43% of oystercatcher feces were positive for the genetic signature of *C. arguinae* by qPCR. Similar prevalence has been observed for other trematodes infecting oystercatchers, such as *C. australis* in New Zealand (Allison, 1979) or *Psilostomum brevicolle* in the Wadden Sea (Borgsteede et al., 1988). Contrastingly, our analyses revealed no infection of gulls. Given that digenean trematodes are commonly less host-specific with respect to their definitive host (Poulin, 2007), gulls could still act as secondary definitive hosts, albeit at much lower prevalence. *C. australis* has indeed been recorded in gulls (*Larus* spp.) (Bennett et al., 2023; McFarland et al., 2003) (Table 7), so any infections present in our study area may simply have been missed. It is possible that the prevalence in all fecal samples was underestimated due to the applied method that is not free of biases. Fecal samples represent a difficult matrix that contains many inhibitors that may have led to reduced genetic detection of *C. arguinae* by qPCR. Also, the number of eggs shed through the bird varies seasonally (according to worm maturity) and even daily (Goater, 1993; Presswell and Lagrue, 2016). As we sampled feces at different times of the day (according to the high tide) and in different months, some fecal samples may not have contained eggs, though the bird was infected by the parasite. Nevertheless, our findings suggest that oystercatchers represent the main definitive host with high prevalence. Adult *C. arguinae* most likely reside in the bird’s gastrointestinal tract, as it is generally the case for himasthlids, such as its congener *C. australis* in oystercatchers in New Zealand (Allison, 1979). This is a commonly infected organ for trematodes with avian definitive hosts, as it provides access to many nutrients and allows the worms to release eggs that are emitted into the environment with the bird’s feces. *C. arguinae* most likely lives in sympatry with other gastrointestinal trematodes (and other helminths). Indeed, oystercatchers (as well as gulls) also serve as definitive hosts to several co-occurring species, such as *Gymnophallus minutus* or *P. brevicolle* (Borgsteede et al., 1988) which also utilize cockles as a second intermediate host.

As few potential eggs were observed in oystercatcher feces, experimentation with different DNA extraction protocols to allow successful molecular identification of eggs was not possible. Trematodes of the genus *Curtuteria* are described as producing few eggs (10–80 per individual) (Odening, 1963; Reimer, 1963; Allison, 1979), which was reported for *C. australis* (Allison, 1979) and seems also to be the case for *C. arguinae*. Though eggs could not be molecularly matched with other life cycle stages directly, the drastically different detection rates in gull vs oystercatcher feces support that the molecular detection was not due to a diet based on cockles infected by *C. arguinae* (as it is also the case for gulls), but rather due to real infection of oystercatchers by adult worms. However, further attempts to optically and molecularly identify eggs of *C. arguinae* are necessary to enable the study of the trematode’s infection phenology at all stages of its life cycle.

## Conclusion

Our study elucidated the complete life cycle of the trematode *C. arguinae*, until now only known to infect cockles as its second intermediate host. The needle snail (*B. reticulatum*) and the European oystercatcher (*H. ostralegus*) were revealed to, respectively, be the first intermediate and definitive hosts of *C. arguinae.* The new identification of these hosts clearly explains why the specific sampling area on Banc d’Arguin was a hotspot of *C. arguinae* infection in cockles. Indeed, the site exhibits favourable conditions for all three hosts. Seagrass patches harbour many needle snails, which are dominant grazers in these habitats, cockles are found buried in and around the seagrass patches in sandy sediments, while oystercatchers visit the area around low tide to prey on various macroinvertebrates. Tidal currents and winds can transport miracidia and cercariae from one host to another throughout the site, facilitating parasite transmission to upstream hosts, most of all cercariae released by needle snails intensely infecting cockles, as it is observed. These findings open the path to investigating the infection phenology at each life cycle stage according to environmental constraints, the degree of the parasite’s pathogenicity for the different hosts and its ecological implications within the marine ecosystem. Our study also highlights the powerful usefulness of eDNA approaches to help detect particularly inconspicuous life cycle stages under challenging conditions. We believe such methods to be particularly well suited for identifying first intermediate hosts which exhibit very low parasite prevalence.
